# Analysis of Cryptic, Systemic *Botrytis* Infections in Symptomless Hosts

**DOI:** 10.3389/fpls.2016.00625

**Published:** 2016-05-10

**Authors:** Michael W. Shaw, Christy J. Emmanuel, Deni Emilda, Razak B. Terhem, Aminath Shafia, Dimitra Tsamaidi, Mark Emblow, Jan A. L. van Kan

**Affiliations:** ^1^School of Agriculture, Policy and Development, University of Reading, WhiteknightsReading, UK; ^2^Laboratory of Phytopathology, Wageningen UniversityWageningen, Netherlands

**Keywords:** gray mold, systemic infection, wild vegetation, *Botrytis*

## Abstract

*Botrytis* species are generally considered to be aggressive, necrotrophic plant pathogens. By contrast to this general perception, however, *Botrytis* species could frequently be isolated from the interior of multiple tissues in apparently healthy hosts of many species. Infection frequencies reached 50% of samples or more, but were commonly less, and cryptic infections were rare or absent in some plant species. Prevalence varied substantially from year to year and from tissue to tissue, but some host species routinely had high prevalence. The same genotype was found to occur throughout a host, representing mycelial spread. *Botrytis cinerea* and *Botrytis pseudocinerea* are the species that most commonly occur as cryptic infections, but phylogenetically distant isolates of *Botrytis* were also detected, one of which does not correspond to previously described species. Sporulation and visible damage occurred only when infected tissues were stressed, or became mature or senescent. There was no evidence of cryptic infection having a deleterious effect on growth of the host, and prevalence was probably greater in plants grown in high light conditions. Isolates from cryptic infections were often capable of causing disease (to varying extents) when spore suspensions were inoculated onto their own host as well as on distinct host species, arguing against co-adaptation between cryptic isolates and their hosts. These data collectively suggest that several *Botrytis* species, including the most notorious pathogenic species, exist frequently in cryptic form to an extent that has thus far largely been neglected, and do not need to cause disease on healthy hosts in order to complete their life-cycles.

## Introduction

*Botrytis* is an ascomycete fungal genus of plant pathogens. Most members of the genus are specialized species infecting a narrow range of monocotyledonous host plants. Typically, they are aggressive, necrotrophic pathogens (Staats et al., 2005). Some have an extended quiescent phase following infection (*Botrytis allii*). An exception to the rule of narrow host range is the clade including the species *Botrytis cinerea sensu lato*. This clade is by far the most economically damaging group within the genus; *B. cinerea s.l*. has a recorded host range including over 1400 (mostly dicotyledonous) hosts (Elad et al., 2015). The typical symptoms leading to economic loss are the occurrence of spreading, fast-growing necrotic lesions bearing abundant pigmented, hydrophobic, conidia.

*Botrytis* conidia are dispersed in windy conditions. Rainfall aids dispersal through the sharp motions of infected tissues resulting from raindrop impact. Conidia require high humidity to infect; infection tests in laboratory are typically performed with high concentrations of spore suspensions (10^6^/ml). The likelihood that conidia will produce a lesion is greatly increased if a nutrient source is available due to host cell damage, insect honeydew or exogenously supplied sugars (van den Heuvel, 1981; Dik, 1992).

Typically *B. cinerea* causes loss in crops by damage to the harvestable part of the crop, flowers, fruits, or leaves, or by girdling stems. The tools to manage *B. cinerea* in crops include (partial) host resistance, avoidance of damage allowing saprophytic infections to occur and then spread, environmental modification to reduce the probability of conditions suitable for spore germination and dispersal, and the use of fungicide. The latter can be effective, but typically there are no consistent critical periods. Although in a single experiment, one or a few fungicide application times may stand out as effective, these application times may not be reproducible in repeat experiments or easy to relate to environmental conditions (e.g., McQuilken and Thomson, 2008). Spores are present in low concentrations in the air over most of the year (Hausbeck and Moorman, 1996; Kerssies et al., 1997; Boff et al., 2001; Boulard et al., 2008). A common strategy for application of fungicides is to maintain continuous cover. This is undesirable on environmental and economic grounds and leads to rapid development of resistance to new fungicides.

A number of publications have suggested that a range of plant species may harbor infections by *B. cinerea s.l*. and other species, including *Botrytis deweyae*, which cause no visible symptoms on the plant at the initial time of infection, are long-lived, can be isolated from newly grown host tissues, but cause necrotic lesions as the plant moves into a reproductive phase. Cultivated hosts in which this form of infection has been studied include *Hemerocallis* (Grant-Downton et al., 2014), hybrid commercial *Primula* (Barnes and Shaw, 2002, 2003), and lettuce (Sowley et al., 2010). In wild-growing plants, Rajaguru and Shaw (2010) found widespread infection in leaves of *Taraxacum* and, to a lesser extent, wild *Primula vulgaris*. A sampling for endophytes in *Centaurea stoebe* revealed the frequent occurrence of *Botrytis* spp., including isolates not derived from known species (Shipunov et al., 2008).

It is becoming increasingly clear that this situation is quite common. A simple distinction between pathogens and non-pathogens may not be possible for many fungal species, as several pathogens have extended cryptic phases (Stergiopoulos and Gordon, 2014). The most obvious examples, and most similar to the *Botrytis* case, are seedborne smut fungi (Schafer et al., 2010), with a cryptic phase from seed until flower maturation. In a more complex example, isolates of *Fusarium oxysporum* can cause devastating disease, with individual fungal genotypes often displaying a very narrow host range or be a benign root endophyte (Gordon and Martyn, 1997; Demers et al., 2015), wherein both endophytic and pathogenic behavior appear to be polyphyletic (Gordon and Martyn, 1997).

The discovery of distributed, symptomless infection by *Botrytis* sp. in a range of hosts suggests that a proportion of inoculum might arise from these sources, and that the fungus may exist for an important part as an endophyte or intimate phyllosphere inhabitant, with conidia only being produced when the host approaches the end of its life-cycle. Understanding this cryptic infection is important for controlling the disease in hosts in which it happens, and for managing disease in other hosts where the cryptic form may serve as an unexpected source of inoculum. In this paper we draw together data on plant species that act as hosts to symptomless distributed infections of *Botrytis*; how the infection varies over time and location; whether particular fungal clonal lineages are adapted to particular hosts; the fungal species involved; the effect of the infection on the host. The aim of experiments collated here was to explore an unexpected mode of infection by a fungus often considered to be a “model necrotroph.”

## Materials and methods

### Surface sterilization

Samples of plant tissues were disinfected with 70% ethanol for 1 min followed by 50% solution of bleach (Domestos, Unilever: 5% NaOCl in alkaline solution with surfactants) for 1 min and rinsed three times in sterile distilled water. In early work, sampled tissues were dipped in paraquat before plating to kill the host tissue and encourage pathogen outgrowth. Seed sterilization was carried out by soaking 0.5 g of seed in 100 ml of 0.10 g/l of the systemic fungicide “Shirlan” (active ingredient 500 g/l Fluazinam, Syngenta Crop Protection UK Ltd.) for 2 h and dried overnight before sowing. This was chosen as the most effective and least phytotoxic of a range of fungicides tested.

### Isolation and culturing, UK

Sampled tissues were placed on plates of *Botrytis* selective medium (Edwards and Seddon, 2001). Samples which turned the medium brown were observed under a dissecting microscope after 12 d exposure to 12 h/day daylight + near UV illumination at 18°C. Fungal colonies growing from the samples and showing the characteristic erect, thick, black conidiophores with *Botrytis*-like conidia were sub-cultured onto malt extract agar.

### Isolation and culturing, Netherlands

*Botrytis*-selective medium was prepared as described by Kritzman and Netzer (1978) with some modifications. The specific composition was (g/l): NaNO_3_, 1.0; K_2_HPO_4_, 1.2; MgSO_4_.7H_2_O, 0.2; KCl, 0.15; glucose, 20.0; agar, 15.0. pH was adjusted to 4.5 and the medium sterilized for 20 min at 120°C. After being cooled to 65°C the following ingredients were added (g/l): tetracycline, 0.02; CuSO_4_, 2.2; p-chloronitrobenzene (PCNB), 0.015; chloramphenicol, 0.05; tannic acid, 5.

### Statistical test of clustering

If the frequency of a tissue sample in plant *i* being infected is *p*_*i*_, the proportion of infected samples on that plant, then a statistic indicating the degree of clustering in a dataset of *n* plants was calculated as

$$\begin{array}{l} \overset{n}{\underset{i}{\sum }}p_{i}\ln\left(p_{i}\right) \end{array}$$

This weights multiple occurrences strongly. The probability of the degree of clustering observed if infected samples occurred independently was judged by repeatedly randomly allocating the total number of positives seen among the total number of samples, grouping the samples into sets representing the individual plants, and recalculating the statistic above. The observed value was compared with an ordered list of the test statistic calculated on the randomizations.

### Sampling sites, host species, and collected plant material

#### England

The Reading University campus, about 130 ha, includes a substantial area of grassland mown annually for hay, mown amenity lawn, formal gardens, woodland, and a lake. Soils are sandy loam overlying river alluvium. Samples of *Taraxacum officinale* and *Bellis perennis* were collected across the campus from mown grassland; *Arabidopsis thaliana* was collected from cultivated beds across the campus and surrounding urban areas; *Tussilago farfara* was collected from lake margins. *Rubus fruticosa agg* and cultivated strawberry were collected from farms near Reading, Brighton and Bath. Further samples of *T. officinale* and *P. vulgaris* were collected from meadow areas near Brighton and Bath.

#### Netherlands

*T. officinale* plants were sampled in four different sites in Wageningen, representing different ecosystems with distinct soil composition. Sampling sites were located around the Wageningen University campus (surrounded by conventional experimental fields and an organic farm with fruit orchards), Binnenveld along the “Grift” canal (boulder clay, grassland with intensive agricultural activities), the floodplain of the Rhine (river clay, grassland grazed by sheep and occasional mowing) and the “Wageningse Eng” (terminal moraine adjacent to the Rhine, loam and sandy soil, recreational use). From each site 25 symptomless, apparently healthy dandelion plants were collected and stored overnight in a cold room until plating, which was done the next day. Plants were cut into three different parts; two leaves, the stem and the flower head. Each tissue sample was surface sterilized by dipping for 30 s successively in 5% bleach, 50% ethanol, and clean water. The leaves, stem and flower head were cut with clean scissors into pieces of 1 cm, of which 3–5 pieces were placed on *Botrytis*-selective medium as described above. Cultures were incubated at room temperature for 6 days. Cultures with the morphological characteristics of *Botrytis* and typical dark-brown color were transferred. A subset of colonies was transferred to fresh medium with lower concentration of CuSO_4_ (2 mg/L), and again incubated at room temperature for 2 days. A *Botrytis*-specific immunoassay (Envirologix, Portland, Maine, USA) (Dewey et al., 2008, 2013) was used to verify that cultures represent a *Botrytis* species.

### Genotyping, gene sequencing, and phylogenetic analyses

Nine microsatellite primers (Bc1–Bc7, Bc9, Bc10) for *B. cinerea* developed by Fournier et al. (2002) were used to genotype isolates randomly sampled from treatments. All isolates were genotyped with all nine primer pairs. The PCR reaction contained 2 μl of water, 5 μl PCR master-mix (Abgene, UK), 1 μl of each forward and reverse primers, and 1 μl of template DNA. The program cycles were: denaturing at 94°C for 3 min, 30 cycles of 94°C for 30 s, annealing temperatures 50°C, (Bc1, Bc3, Bc6, and Bc9), 53°C (Bc2 and Bc5), or 59°C (Bc4, Bc7, and Bc10), and 72°C for 30 s.

DNA isolation and the amplification of fragments of three house-keeping genes (Heat Shock Protein 60, HSP60; Glyceraldehyde 3-Phosphate Dehydrogenase, G3PDH; DNA-dependent RNA Polymerase subunit II, RPB2) were performed as described by Staats et al. (2005). Amplification of three additional genes (G3890, FG1020, MRR1) was performed based on sequences reported by Leroch et al. (2013). Gene fragments were sequenced by Macrogen (Amsterdam, Netherlands), and subsequent phylogenetic analysis was performed as described by Staats et al. (2005).

All primers used for the amplification of microsatellite markers or genes for sequencing are listed in Table S1.

### Specific experimental designs

#### Experiment 1: Cryptic systemic infection in *Arabidopsis thaliana*

*A. thaliana* plants were grown in a filtered air flow supplied to individually covered pots in a CE room, with a 16 h light and 8 h dark period. Inside the covers, day-time temperature was 26.5°C and night 18.5°C; relative humidity in day and night ranged between 80 and 85%. Light intensity was 200–250 μmol/m^2^/s. The plants were watered from below so as to keep the compost just moist: every day up to 2 weeks from sowing then at two-day intervals. Spores were collected from plates of *B. cinerea* B05.10 using a cyclone collector and serially diluted in talc powder through five stages, each by a factor of 10. Five milligrams of diluted spore dust was dusted on plants 21 days after sowing using a porthole in the top of the plant cover, otherwise kept covered with sticky tape. Controls were dusted with talc powder only. Ten replicates were maintained for each treatment and control. Sampling of plant tissues was done on BSM plates 10 days after inoculation. From each plant, two stem segments (~2 cm), three rosette leaves, two stem leaves, a piece of root (~2 cm long), and two inflorescences were separately sampled as surface disinfected and non-surface disinfected.

#### Experiment 2: Pathogenicity and host specialization

(A) Isolates ES13 (from lettuce), Gsel G07 (from *Senecio vulgaris*), Arab A07 (from wild *A. thaliana*), and Dan D07 (from *T. officinale*) were inoculated onto detached leaves of *A. thaliana, T. officinale, S. vulgaris, T. farfara*, and lettuce by placing a 5-mm agar plug of the fungal culture grown on PDA on the adaxial side of a surface sterilized leaf of the host to be tested. The leaf was placed on damp filter paper in 20 × 10 cm plastic boxes with the lid covered. A plug of PDA was used as a control. Ten replicates were made; a single box contained only one type of leaf. Sizes of the necrotic lesion on the leaves were measured after 5 days, as the maximum dimension on asymmetric leaves. Data were analyzed by analysis of variance allowing for the split-plot design. (B) Conidia were harvested from 8 to 10 day old cultures grown on PDA and suspended in sterile water with 0.01% Tween-20 solution, separated by vortex-mixing and adjusted to 1000/μl. Droplets of 5 μl were inoculated on 1 cm^2^ leaf pieces cut from leaves of lettuce, *S. vulgaris, T. farfara*, and *T. officinale*. Infected leaf pieces were counted after 5 days. Results were analyzed using a generalized linear model with Bernouilli error and a logistic transform.

#### Experiment 3: Pathogenicity and host specialization

Detached leaves of tomato (cv. Moneymaker) or leaves of whole plants of *Nicotiana benthamiana* and *T. officinale* (grown from surface-sterilized seeds) were inoculated with 2 μl droplets of conidial suspensions of different *Botrytis* isolates (10^6^/ml in Potato Dextrose Broth, 12 g/l). Inoculated plant material was incubated in a plastic tray with transparent lid at 20°C in high humidity. Disease development was recorded on consecutive days from 3 to 7 days post inoculation. The proportion of inoculation droplets clearly expanding beyond the site of inoculation was taken as a measure for disease incidence (in %), the diameter of an expanding lesion (in mm) was taken as a measure for disease severity.

#### Experiment 4: Sporulation and transmission in lettuce

Fungicide seed sterilization was carried out by soaking 0.5 g of seeds in 100 ml of (0.10 g/l) systemic fungicide “Shirlan” (Syngenta) for 2 h and drying overnight before sowing. Seedlings were transplanted to 1 L pots when three true leaves were emerged; in half the pots ten 1 cm plugs of a *Trichoderma harzianum* T39 culture were placed in the compost at transplanting. A suspension of *Botrytis* ES13 (10^5^ spores/ml) was sprayed onto half the plants 4 weeks after transplanting. Plants were covered with polythene bags for 24 h to retain high humidity following inoculation. Seed treated and untreated plants were arranged in randomized blocks each containing a replicate of the experiment (±seed sterilization × ±*Botrytis* inoculation × ±*Trichoderma* inoculation). At 14 weeks, plants were harvested and samples plated on BSM. Five 1-cm diameter leaf disks from five different leaves, five samples scraped from inside the stem, and five 1-cm long pieces of tap and secondary root system were randomly selected from each test plant. All samples were from healthy tissue with no visual sign or symptoms of damage. Samples were washed under running tap water, then surface sterilized, and placed on BSM to detect *Botrytis*, as described above.

#### Experiment 5: Transmission in dandelion

Forty non-sterilized and 40 surface-sterilized seeds of *T. officinale* (sampled on the river banks of the Rhine) were grown on BSM for 3 days to test whether they were infected by *Botrytis*. Seeds were then transferred to wet filter paper for a week and 20 selected germinated seedlings were transferred into an autoclaved sand-soil (1:1) mixture. The plants were grown in separate plastic pots for 2 months until they were of sufficient size for infection assays. Symptomless *Botrytis* infection in *T. officinale* leaves was monitored before the infection assay was performed. One leaf of each plant was selected, surface sterilized and cultured on a BSM plate. *Botrytis* outgrowth was evaluated by checking the color change in the medium and subsequent sporulation.

#### Experiment 6: Effects on host and environmental interactions

Commercially produced lettuce seed cv. “All the Year Round” was germinated in large seed trays. Two weeks later, seedlings were transplanted to 1 L pots of John Innes 2 compost. Thirty seedlings were tested for infection by *Botrytis* as before; no infection was found. Pots were grouped in sets of four, inoculated or not. A 2^6^ factorial design with treatments inoculation × temperature × shading × nitrogen form was then laid out with six randomized blocks of inoculation × shading × nitrogen form in each glasshouse (192 plants in total). Groups of four pots were shaded using green mesh approximately 30 cm above the pots, draped down the sides. Pots were fertilized twice weekly with either 4 mM KNO_3_ or 4 mM (NH_4_)_2_SO_4_ +2 mM K_2_SO_4_. Thirty-six days after sowing, the pots to be inoculated were moved to a separate glasshouse. They were arranged in trays of 16 and inoculated with dry spores by tapping a plate above the tray, followed by enclosure in black plastic for 30 min to allow spores to settle. At 1, 2, and 3 months after sowing one plant was removed from each set of four and dissected to give 12 tissue samples: edge and central samples from an old leaf, a mature leaf, and a young leaf, three stem and three root samples. These were plated on BSM as before and the presence of *Botrytis* noted. At the third harvest, both remaining plants were cut at the base and oven-dried before weighing. The weight of the tissues that were removed to test for the presence of *Botrytis* was negligible. Temperature effects are confounded with glasshouse block effects in this design, but this was unavoidable; differences in nitrogen form are also confounded with sulfate supply, also this was unavoidable. The two compartments had the same aspect, shape and shading patterns, so the largest single difference was temperature.

## Results

### Host range and prevalence

*Botrytis* species were frequently isolated from the interior of multiple tissues in apparently healthy hosts of many species (Table 1). Prevalence reached 50% of samples or more (as in *T. officinale*), but was usually less, and cryptic infections other than in flowers were rare or absent in *T. farfara, B. perennis*, or *P. vulgaris*. Prevalence varied substantially from tissue to tissue. Species in which fruit infection is frequently reported did not necessarily harbor latent infection in other tissues of the plant: in *R. fruticosa* agg. and cultivated strawberries *Fragaria* × *ananassae*, leaf infection was not found. There was no clear association with particular plant families in the small sample represented here, nor with perenniality. Repeated surveys are available for the perennial herb *T. officinale* and the short-lived annual *A. thaliana*. Prevalence varied substantially from year to year, from place to place and from tissue to tissue (Table 2).

**Table 1:** **Percentage of samples showing cryptic infection with ***Botrytis*** in various host plant species**.

**Family**	**Host species**	**Growth form**	**Growing situation^a^**	**Location^a^**	**Sample size**	**Flower sample size^b^**	**Organ isolated from**				**Randomly associated?**
							**Fruit or petals**	**Leaf**	**Stem**	**Root**	
Asteraceae	*Tussilago farfara*	Perennial cryptophyte	Lake edge	RU	45	10	0^c^	0	0	2	n/a^d^
	*Bellis perennis*	Perennial herb	Lawn	RU	55	21	0	0	3	0	n/a
	*Gerbera x hybrida*	Perennial herb	Indoor crop	RU	96	96	0	0	0	0	n/a
	*Taraxacum officinale complex*	Perennial herb	Open grassland, lawn	RU	107	75	27	29	21	16	*P* < 0.001
				WU	100	100	72	87	72	–^c^	n.t.^e^
	*Cirsium vulgare*	Perennial herb	Open grassland	RU	24	18	2	4	1	0^c^	*P* > 0.5
	*Senecio vulgaris*	Ruderal	Cultivated ground	RU	82	62	31	37	26	9	*P* = 0.002
	*Centaurea scabiosa*	Perennial cryptophyte	Wild	RU	35	23	22	49	23	21	*P* = 0.6
	*Achillea millefolium*	Perennial herb	Open grassland	RU	44	35	56	32	16	11	*P* = 0.002
Brassicacae	*Arabidopsis thaliana*	Short-lived annual herb	Wild	RU	66	41	30	48	41	5	*P* < 0.001
Primulacae	*Primula vulgaris*	Perennial herb	Wild	SE	382	–	–	8	–	–	n/a
Rosaceae	*Potentilla fruticosa*	Perennial shrub	Landscape planting	RU	100^f^	–	–	8	7	–	*P* < 0.001
	*Rubus fruticosa agg*.	Perennial climber	Hedges	SE	219	219	101	0	0	0	n/a
	*Fragaria × ananassae*	Perennial herb	Crop	SE	203	203	77	0	0	0	n/a

a *RU: grounds of Reading University, UK; SE: several locations across southern England; WU, four locations around Wageningen, NL*.

b *Fruiting structures were not necessarily present in all plants sampled*.

c *0: no infection found; – not sampled*.

d *Test not applicable: infestation found in only one organ per plant*.

e *Not tested*.

f *Three bushes, samples 40, 40, 20. All infections came from the bush with sample size 20*.

**Table 2:** **Percentage of tissue samples of wild-growing ***Taraxacum officinale*** complex and ***Arabidopsis thaliana*** from which ***Botrytis*** could be isolated after surface sterilization, at different locations and years**.

**Species**	**Year**	**Location**	**Source**	**Root %**	**Leaf %**	**Stem %**	**Flower %**	**Sample size**
*T. officinale*	2005–6^a^	Reading	Cooray	5	6	–^b^	–	100
	2005–6^a^	Bath	Cooray	13	5	–^b^	–	110
	2005–6^a^	Brighton	Cooray	0	4	–^b^	–	132
	2007–8^a^	Reading	Shafia	18	29	–^b^	27	108
	2008	Reading	Thriepland	17	31	–^b^	–	(182)^c^
	2014	Reading	Emblow	0	1	–^b^	6	24
	2014	Wageningen	Onland and Hoevenaars	–^b^	87	72	72	100
*A. thaliana*	2007–8^a^	Reading	Shafia	5	50	41	29	66
	2010	Reading	Shaw	10	20	0	0	20
	2013	Reading	Emmanuel	4	2	3	3	76
	2014	Reading	Emmanuel	0	0	0	5	22
	2014	Reading	Emblow	0	0	0	0	31

a *Data were sampled separately in autumn, spring and summer; proportions of samples infested were homogenous (χ^2^ P > 0.2)*.

b *–, not sampled*.

c *From nine seedling families. There were no significant differences between families (χ^2^-test, P = 0.2)*.

### Evidence for spread in and over a single host: Experiment 1

**Figure 1:**
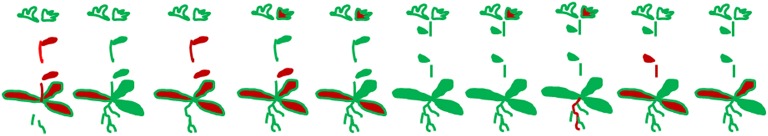
**Clustering of recovery of ***B. cinerea*** B05.10 from surface sterilized tissues of ***Arabidopsis thaliana*** grown in a sterile airflow and inoculated with dry spores at the two leaf stage**. Each drawing of a plant represents the samples into which it was dissected. *B. cinerea* was recovered from samples colored red; note the strong clustering of infected samples in individual plants (*P* < 0.001).

Published evidence about the spread of *Botrytis* in a single host is available for cultivated hybrid *Primula* (Barnes and Shaw, 2003) and lettuce (Sowley et al., 2010). In the majority of host species in which isolations could be made from distinct tissues, multiple isolations from a single plant were more common than expected (Tables 1, 2). *A. thaliana* that were grown in a sterile air-stream and inoculated as seedlings with dry conidia at low density developed normally and did not show any disease symptoms or evidence of stress. *Botrytis* could be isolated from multiple tissues, many of which only developed after the time of inoculation (Experiment 1). Isolations were strongly clustered in individual plants, consistent with systemic spread in the plant (Figure 1; randomization test *P* < 0.001).

In *Cyclamen persicum*, inoculation of four plants with a spore suspension followed by enclosure in a plastic bag led to serious necrosis development with abundant sporulation. The plants recovered upon removal of the bags and produced new flushes of leaves and eventually flowered, without further signs of *Botrytis*. Of 14 seed capsules examined (381 seeds) one capsule yielded two *Botrytis* infected seeds (out of 47) and a second capsule yielded 56 *Botrytis* infected seeds (out of 57). The seeds from the other 12 capsules were completely free of *Botrytis*. Isolations were attempted from leaves, roots, exterior and interior samples from the corms. *B. cinerea s.l*. was isolated from roots of two plants and the exterior and interior of the corm of two others (Table 3).

**Table 3:** **Recovery of ***Botrytis*** from different tissues of four ***Cyclamen persica*** plants cv. Midori earlier inoculated and diseased, but recovered and symptomless at the time of sampling**.

**Plant**	**Root**	**Flower pedicel**	**Petal**	**Leaf**	**Outer corm**	**Inner corm**
Midori White (plant #1)	1	1	1	0	0	0
Midori White (plant #2)	0	1	0	0	1	0
Midori Scarlet (plant #1)	1	1	0	0	0	0
Midori Scarlet (plant #2)	0	1	0	0	0	1

### Phylogeny and species variation of internal infections in dandelion

**Figure 2:**
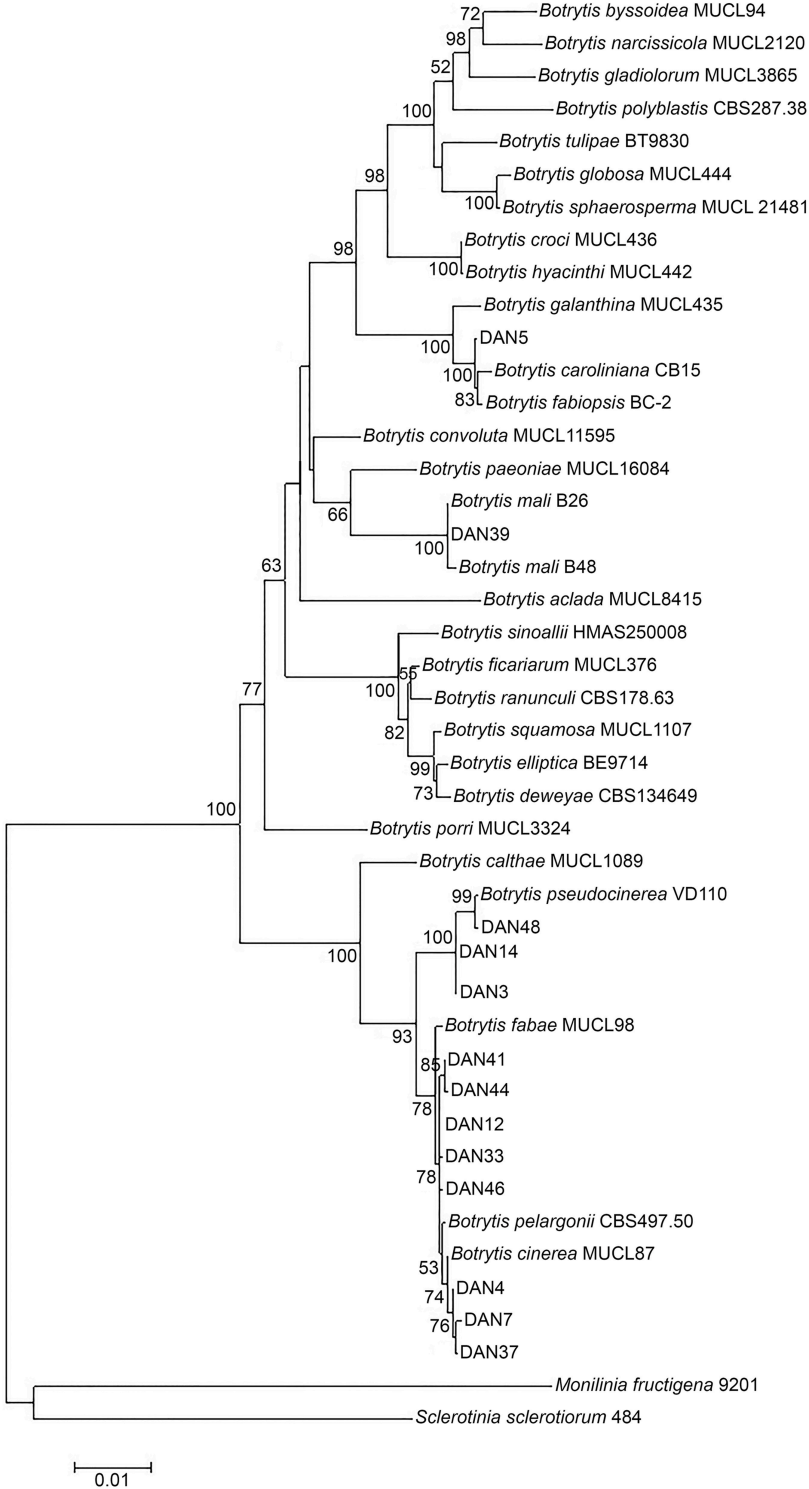
**Phylogenetic position of 13 ***Botrytis*** isolates from asymptomatic dandelion (labeled by DAN and a number) in the genus based on concatenated HSP60, G3PDH, and RPB2 sequences**. Recognized *Botrytis* species are taken from Hyde et al. (2014). Phylogenetic tree construction used a maximum likelihood method with 1000 bootstraps. Only bootstrap values higher than 50% are displayed at the nodes.

We aimed to test whether strains of *Botrytis* developing cryptic asymptomatic infection form a separate phylogenetic group, distinct from strains that cause necrotic infections. A preliminary phylogenetic clustering of 47 *Botrytis* isolates sampled from asymptomatic dandelion in Netherlands was performed based on HSP60 and G3PDH gene sequences (not shown). Thirty-five out of forty-seven isolates grouped with the *B. cinerea* species complex and 10 grouped with *Botrytis pseudocinerea*, all members of clade 1 in the genus *Botrytis* (Staats et al., 2005). Two other isolates grouped into clade 2, i.e., isolates DAN5 and DAN39. Based on this result, eight isolates grouping with *B. cinerea* and three isolates grouping with *B. pseudocinerea*, together with the unknown isolates DAN5 and DAN39, were selected for additional analysis of the RPB2 gene sequence. Using as backbone the multiple sequence alignment of 29 known species of the genus *Botrytis* (Hyde et al., 2014), the concatenated sequences of three genes (HSP60, G3PDH, and RPB2) from the 13 isolates sampled from asymptomatic dandelion were included for phylogenetic tree construction (Figure 2). Eight isolates were related, but not identical, to *B. cinerea* type isolate MUCL7. Three isolates were related but not identical to *B. pseudocinerea* isolate VD110. Isolate DAN5 clustered with, but is not identical to, *Botrytis caroliniana* and *Botrytis fabiopsis*, while DAN39 clustered very tightly with two *Botrytis mali* isolates and is probably a member of this species. There was good agreement between phylogenetic trees for individual gene sequences HSP60, G3PDH, and RPB2 (Figures S1–S3). In order to provide a better phylogenetic resolution of isolates that were most closely related to *B. cinerea*, 16 isolates were analyzed in more detail for the sequences of three additional genes (G3890, FG1020, MRR1; Figures S4–S6). The combined phylogeny for these three genes (not shown) revealed that nine of the 16 isolates were closely related to *Botrytis* type S, a subgroup of isolates within the *B. cinerea* complex that was initially predominantly sampled from strawberry and is typified by a characteristic insertion of 21 nucleotides in the MRR1 gene (Leroch et al., 2013). The other seven isolates were closely related to *B. cinerea* but distinct from both *B. cinerea* groups N and S (not shown).

### Mating type analysis

To investigate the mating type of the 47 isolates sampled from asymptomatic dandelion in Netherlands, PCR reactions were carried out that amplify a gene in the MAT locus (Amselem et al., 2011). Both mating type alleles (MAT1-1 and MAT1-2) were found in similar proportions in the isolates from the *B. cinerea* species complex and from *B. pseudocinerea*. In both cases the mating types were present in close to a 1:1 ratio. This observation does not necessarily imply that the isolates are progeny from sexual reproduction but at least they have the possibility of finding compatible mating partners within the same host species. Isolate DAN5 has mating type MAT1-1 and isolate DAN39 has mating type MAT1-2 (Table 4).

**Table 4:** **Distribution of mating types in 47 ***Botrytis*** isolates from dandelion, as determined by diagnostic PCR**.

**Species**	**Mating type MAT1-1**	**Mating type MAT1-2**
*B. cinerea*	19	16
*B. pseudocinerea*	5	5
DAN5, related to *B. caroliniana* and *B. fabiopsis*	1	–
DAN39, putative *B. mali*	–	1

### Pathogenicity and host specialization

If isolates from asymptomatic plants are distinct from pathogenic strains and unable to cause disease, they would not pose a threat to neighboring plants and especially crops. If such isolates, however, have the capacity to cause necrotic symptoms (given the right circumstances), they could at some point in time strongly increase the risk of disease development, either in the symptomless host, or in neighboring (crop) plants. In the next two experiments we therefore aimed to test the capacity of *Botrytis* isolates from symptomless plants to cause necrotic symptoms on host species from which they were recovered, as well as on other plant species. Artificial inoculation under laboratory conditions requires a suitable spore density, availability of nutrients, and proper environmental conditions to accomplish necrotic infection. We therefore used standard methods with either mycelium on agar plugs or spore suspensions as inoculum.

#### Experiment 2

**Figure 3:**
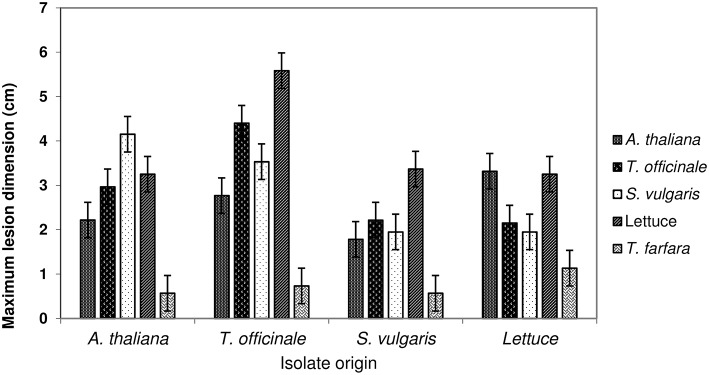
**Lesion diameters 5 days after agar plug inoculations of isolates of ***Botrytis*** sampled from four hosts onto entire detached leaves of ***Arabidopsis thaliana***, ***Taraxacum officinale, Senecio vulgare***, cultivated lettuce, and ***Tussilago farfara*****. Error bars represent 1 *SED*; non-overlapping bars are different at *P* = 0.05 (uncorrected for multiple comparisons).

Isolates sampled from five different host species (*A. thaliana, T. officinale, S. vulgaris, T. farfara*, and lettuce; one isolate per host origin) were inoculated on newly grown, asymptomatic plants of four host species (the same as above with the exception of *T. farfara*) in all possible combinations. Inoculations were performed with cultures grown on an agar plug and lesion sizes were monitored (Figure 3). In this test there were differences in virulence (*P* < 0.001) and in host susceptibility (*P* < 0.001) and a significant (*P* = 0.001) but minor interaction between host susceptibility and pathogen virulence in both cases. There was no tendency for isolates to be more pathogenic or less pathogenic on their host of origin. Results were similar when inoculation was performed with spore suspensions.

#### Experiment 3

**Figure 4:**
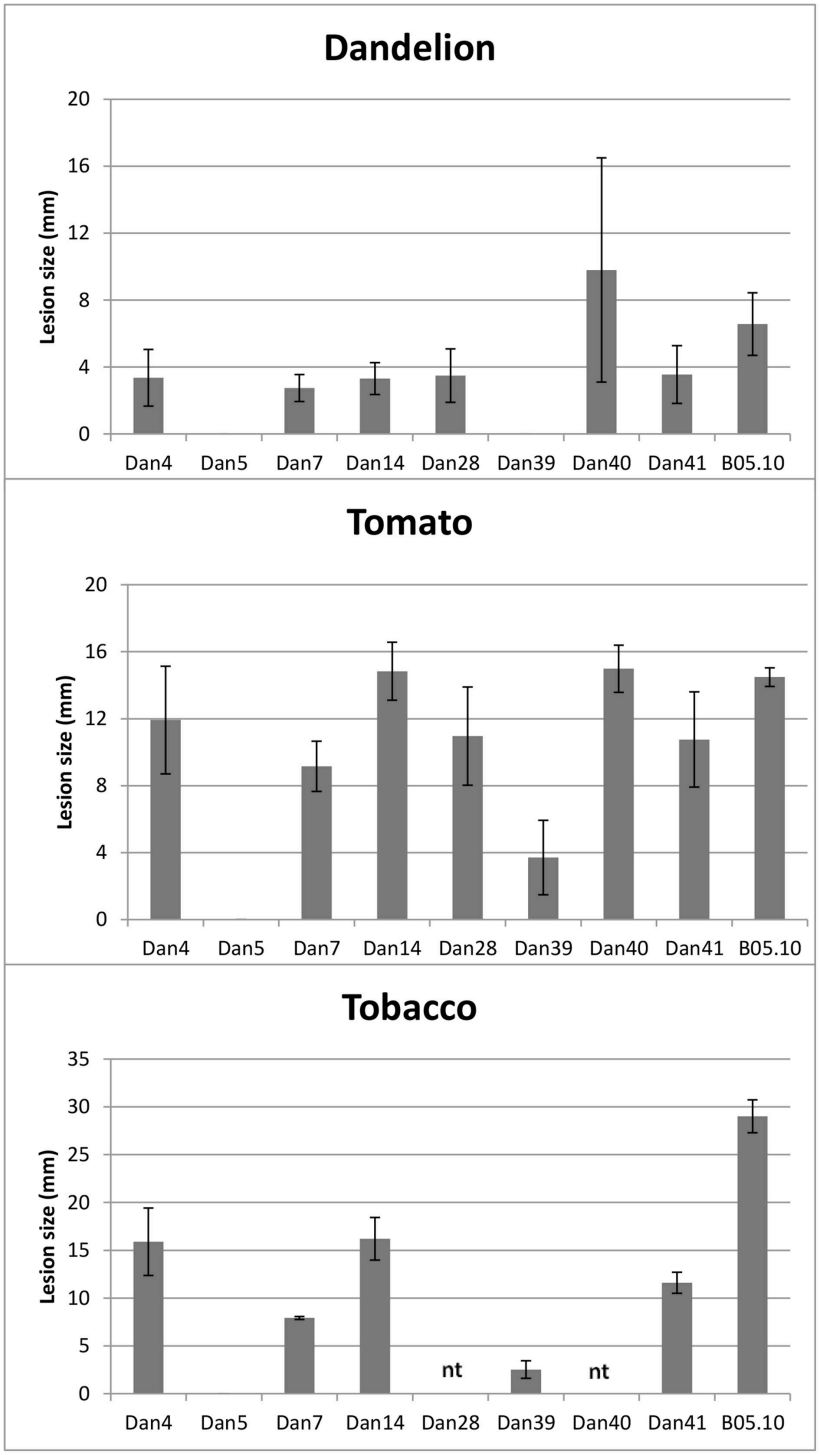
**Lesion diameter on leaves of ***Taraxacum officinale*** at 5 days after inoculation (***N*** = 6), tomato at 3 days after inoculation (***N*** = 8) and ***N. benthamiana*** at 3 days after inoculation (***N*** = 3), following inoculation with ***Botrytis*** isolates or ***B. cinerea*** strain B05.10**. Data presented are means with standard deviations indicated with the error bars.

Eight *Botrytis* isolates sampled from asymptomatic dandelion in Netherlands were selected for artificial infection assays on leaves of tomato, *N. benthamiana* and *T. officinale* plants. Three isolates were selected from the *B. cinerea* species complex, three were from *B. pseudocinerea* and the remaining two were isolates DAN5 (related to *B. caroliniana* and *B. fabiopsis*) and DAN39 (putative *B. mali*). As a reference, the commonly aggressive *B. cinerea* strain B05.10 was always inoculated on the opposite leaf half. Results of these infection experiments are shown in Figure 4. The *B. cinerea* and *B. pseudocinerea* isolates all produced expanding lesions on leaves of tomato and *N. benthamiana*, disease incidence was 100%. On *T. officinale* leaves, disease incidence for all isolates from asymptomatic dandelion was below 40%, while for *B. cinerea* strain B05.10 it ranged from 60 to 90%. Isolate DAN5 was entirely unable to cause disease on the three hosts tested. At most it caused small black dots at the inoculation sites, which never developed into expanding lesions at later time points. Isolate DAN39 was able to cause expanding lesions on tomato and *N. benthamiana*, but not on *T. officinale* leaves. The lesion diameters differed between isolates and hosts. In general, the diameters of lesions caused by *B. cinerea* and *B. pseudocinerea* isolates on tomato leaves were similar to those of strain B05.10, while on *N. benthamiana* leaves, the lesions were smaller than those of B05.10 (Figure 4). Symptoms on *T. officinale* leaves were generally few and mild. *B. pseudocinerea* isolates sampled from symptomless dandelion differed in disease severity on dandelion, the most virulent isolate (DAN40) caused lesions of similar size as those caused by *B. cinerea* B05.10 whereas the two others (isolates DAN14 and DAN28) caused lesions quite smaller than those of B05.10 (Figure 4). The experiments above show that *Botrytis* isolates sampled from asymptomatic plant species have the capacity to cause necrotic lesions on the hosts from which they were sampled, as well as on distinct, unrelated plant species.

### Sporulation, vertical transmission, and closure of the life-cycle

The following experiments were designed to examine whether cryptic infections can be long-lived within a plant and can pass from seeds to mature plants.

#### Experiment 4, lettuce

This experiment was intended to test the hypothesis that cryptic infection would reduce host growth, and that competing endophytes would reduce this effect. A batch of lettuce seed was used from plants grown outdoors and displaying a 98% frequency of seed infection with *B. cinerea*. A randomized block full factorial experiment was done with treatments combining seed disinfection with fluazinam, spray inoculation with *B. cinerea* spore suspension 4 weeks after transplanting (approximately at 10 leaf stage) and compost inoculation with a *T. harzianum* T39 isolate thought to be a possible biocontrol agent for the internal *Botrytis*. There were no significant differences in the proportion of plant samples from which *Botrytis* was recovered (28 ± 3%), so the hypothesis could not be assessed. Only two of the 17 plants from which isolated *Botrytis* cultures were further characterized displayed symptoms of *Botrytis* infection. Treatment with *T. harzianum* neither had a detectable impact on the colonization of the lettuce plants by *Botrytis* nor on the genotypes recovered from plants exposed to *T. harzianum*.

To further investigate the results, microsatellite haplotypes were determined from 15 isolates from different tissues of fungicide treated plants and from 13 isolates from untreated plants (Table 5). All the isolates from fungicide *untreated* plants were identical to each other (haplotype B), but distinct from the isolate used for inoculation (haplotype C). The fungicide *treated* plants contained six different haplotypes including three recoveries of the inoculated isolate (haplotype C) and a single representative of haplotype B, characterizing the fungicide *untreated* plants (Table 5; randomization test *P* < 0.001 against the null hypothesis of random recovery).

**Table 5:** **SSR genotypes, based on eight loci, of ***Botrytis*** isolates recovered from a randomized block factorial experiment on lettuce with treatments of fungicide seed treatment, spray inoculation with isolate ES13 (haplotype C) at the 4-leaf stage and soil inoculation with ***T. harzianum T39*****.

**Fluazinam**	***B. cinerea inoculation***	***T. harzianum* T39 inoculation^a^**	**Plant ID**	**Root**	**Stem**	**Leaf**
+	+	−	6			C, C
			7		E	
			17		E^b^	D,D^b^
	−	+	8	F		
			13			B
			16		A	
		−	3			C,F
			4	A		
			5		A	
−	+	+	2			B,B,B,B
			9		B,B	
		−	1			B,B,B,B
			14		B	
	−	+	11		B	
			15			B
		−	10			B
			12		B	

*Letters A–H represent distinct haplotypes found in the Botrytis strains isolated from the host tissue samples*.

a *No samples from the fungicide + T. harzianum + Botrytis inoculation were genotyped*.

b *Symptomatic at time of sampling*.

This observation suggests that a pre-existing cryptic infection originating from a seed-borne isolate of *Botrytis* excluded further infection until an advanced phase of plant growth, and that the level of internal infection sustained was similar regardless of the source of inoculum.

#### Experiment 5, *Taraxacum officinale*

Two batches of dandelion plants were grown, one from non-sterilized seeds and the other from surface-sterilized seeds. Placing untreated seeds directly on selective medium established that *Botrytis* cultures grew out from 100% of the seeds. Both batches of dandelion plants were symptomless throughout their growth. There was no significant difference in the number of leaves or leaf lengths between plants grown from surface-sterilized seeds and the plants grown from untreated seeds (not shown). *Botrytis* infection in leaves was investigated after 2 months of growth, when plants reached maturity. *Botrytis* infection was observed in four out of 20 leaves from dandelion plants derived from non-sterilized seeds in the first trial, and 1 out 17 leaves in the second trial. All the plants derived from surface-sterilized seeds were free of *Botrytis*. The difference is significant at *P* = 0.02. Therefore, in dandelion, seed infection can survive and grow throughout a proportion of plants until flowering produces the next generation of seed.

### Effects on the host and environmental interactions: Experiment 6

Cryptic infection might impose a defensive load on a host plant. This experiment was designed to test whether factors tending to improve host growth, and thereby reducing resources available for defense, would reduce infection. To measure the effect of cryptic *Botrytis* infection on growth, commercial seed with a low level of *Botrytis* contamination was used and half of the plants deliberately dusted with *Botrytis* spores. Temperature, fertilization and light were varied to produce different growing conditions.

**Figure 5:**
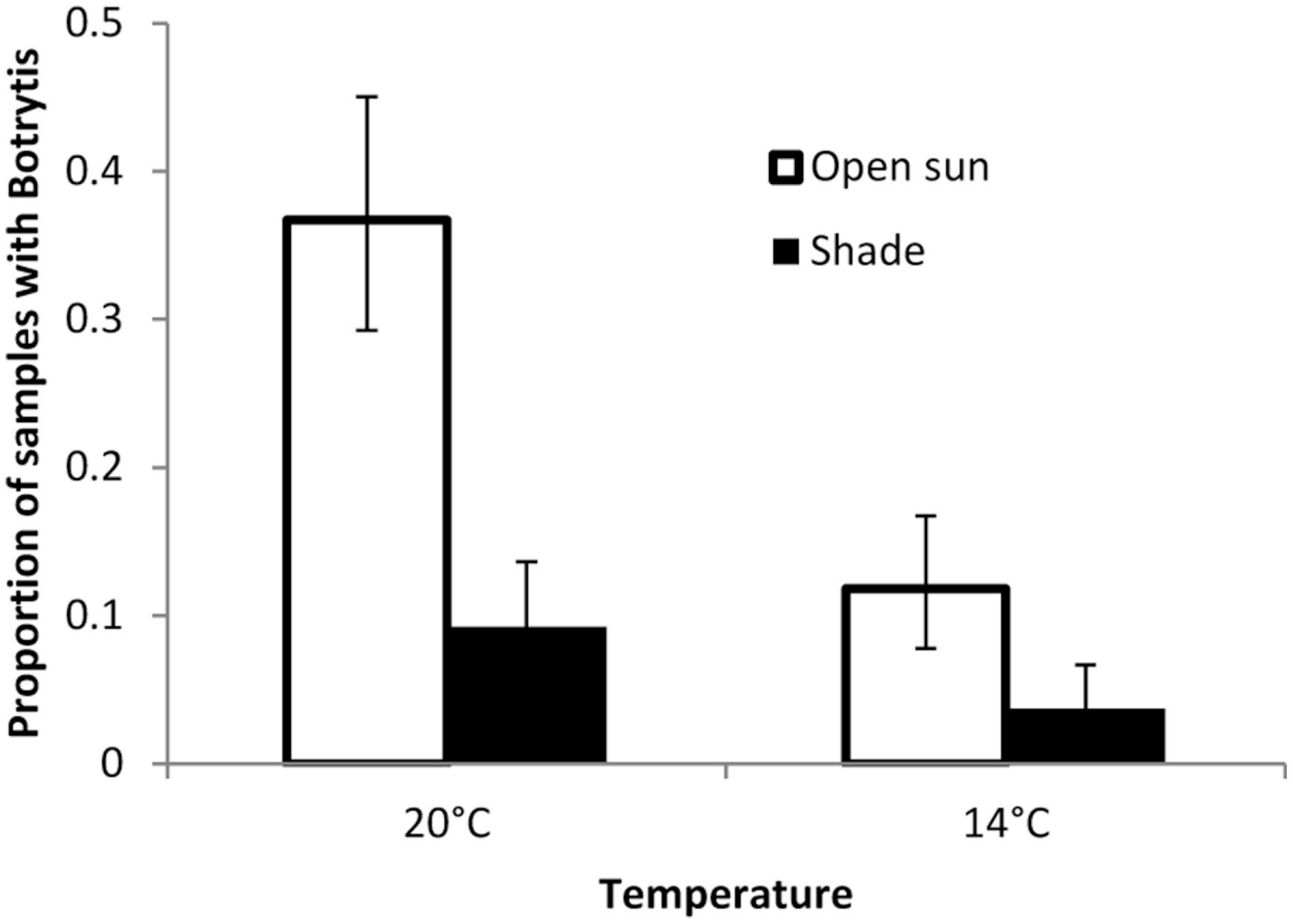
**Proportion of samples of lettuce plant tissue from which ***Botrytis*** isolates could be recovered after surface sterilization when grown in shaded or open positions in glasshouse conditions**. Back-transformed data from anova on square root transformed data. Error bars are 1 *SED*.

Inoculation of lettuce with *Botrytis* spores not leading to necrotic infection did not result in changes in the frequency with which *Botrytis* could be isolated from the plants (factorial anova *P* = 0.8). Infection frequencies were approximately doubled in unshaded plants compared to plants grown in shaded conditions, and approximately doubled in plants grown at 20°C compared to plants grown at 14°C (Figure 5). Ammonium and sulfate fertilization gave about a 10% relative increase in infection over nitrate fertilization (*P* = 0.07).

**Figure 6:**
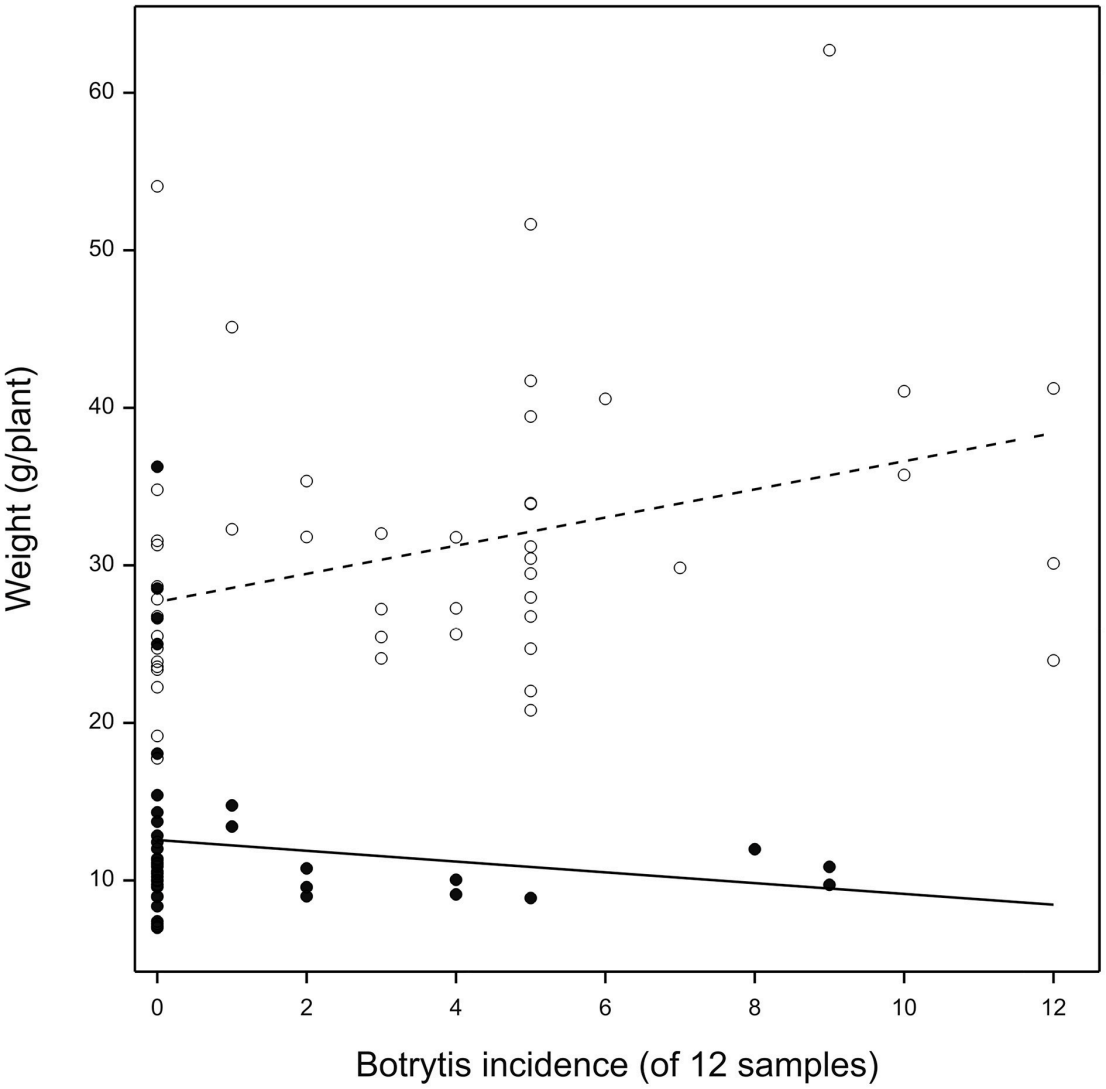
**Dry weight at harvest (g) of lettuce plants grown in shaded (closed circles) or unshaded (open circles) glasshouse conditions in relation to the number of tissue samples (of 12) from each plant in the experiment (***n*** = 96) from which ***Botrytis*** isolates could be recovered after surface sterilization**.

At final harvest, the *Botrytis*-inoculated plants were 19% lighter in weight than the uninoculated plants (anova *P* = 0.02) in unshaded conditions and 23% lighter in weight (anova *P* = 0.05) in shaded conditions. This result was quantitatively reproducible. The relation between the weight of a lettuce plant and the proportion of *Botrytis*-infected surface sterilized samples from the same plant depended on whether plants were shaded or unshaded (Figure 6). In unshaded plants there was a weak *positive* relation between cryptic infection and weight (+ 0.3 percentage points per 1 percentage point increase in infection, *P* = 0.01). In shaded plants there was a weak and non-significant negative relation (−0.3 percentage points per 1 percentage point increase in infection, *P* = 0.2).

This experiment establishes that cryptic infection was not limited by inoculum supply (confirming experiment 4), that conditions favoring host growth, especially light, also favored cryptic infection, and that *Botrytis* inoculation, despite not altering the final levels of infection, was strongly detrimental to growth.

## Discussion

Specialist *Botrytis* species such as *B. aclada* and *B. allii* have been understood to have a life-cycle involving extended periods of symptomless growth (Maude and Presly, 1977; du Toit et al., 2004). Seed infection by species in the *B. cinerea* complex leading to seedling infection has been well-known for some time in species such as linseed (Harold et al., 1997) because of its contribution to seedling death. It has been less appreciated that long-lived cryptic infection with generalist *Botrytis* is possible. This has previously been shown in cultivated primula (Barnes and Shaw, 2003) and in lettuce (Sowley et al., 2010). Quiescent infections of a few host cells which give rise to spreading necrosis at fruit maturity are characteristic of many fruit diseases (e.g., Prusky and Lichter, 2007; Puhl and Treutter, 2008). The mode of growth discussed here is different, as it is a form of infection in which the fungus is able to grow with the host and spread to new plant parts. The results above show that many host species growing in natural settings and showing no disease symptoms frequently have disseminated infections of *Botrytis* species.

This is of both scientific and practical interest. We become increasingly aware that plants harbor many endophytic species (e.g., Shipunov et al., 2008) and that these may have profound effects on their susceptibility to stress and to pathogens (Rodriguez et al., 2009). Understanding that a pathogen like *Botrytis*, which is investigated mostly in the context of rapidly spreading necrotic infections may also exist as a widespread symptomless infection with minimal effects on the host should seriously alter our understanding of pathogenesis. There are quantitative differences between isolates in their pathogenicity on different species, but we did not obtain any evidence for isolates taken from a particular host to be particularly adapted to that host. Furthermore, both *B. cinerea, B. pseudocinerea*, and other lineages or cryptic species of *Botrytis* appeared to occur in this asymptomatic form. From a practical point of view, management of *Botrytis* in species where cryptic infection is common needs to focus on environmental conditions; it may be ineffective to try to prevent low levels of infection, since permanent cover by systemic fungicide would be needed. In particular, results in lettuce suggest a balanced system in which host defenses are activated sufficiently to prevent infection increasing above a certain density, because plants grown from “clean” seed acquired the same level of infection as plants grown from the infection-carrying seed infection. The question whether cryptic infection is harmful or beneficial to a plant is difficult to address because of the difficulty of growing plants free from *Botrytis* infection but also in a natural setting.

Because the findings reported may be deemed somewhat surprising, it is important to consider possible artifacts. The most obvious is that these isolations could represent independent, spatially restricted early stages or quiescent infections from environmental conidia. There are several arguments against an important contribution of these artifacts to the observations summarized here. (1) Barnes and Shaw (2003) showed that the sterilization procedure was highly effective in killing adherent spores on seed and other tests with inoculated material have shown the procedure to be effective on leaf tissue. (2) *T. officinale* and *B. perennis* grow in the same habitat and often in close proximity but have very different prevalence rates, showing that we are dealing with at least an established association between fungus and host. (3) In greenhouse work, Barnes and Shaw (2003) showed that the same isolate was repeatedly recovered from single hosts, but that adjacent plants rarely hosted identical isolates. This is weak evidence by itself, but supports the hypothesis that this type of infection is at least partially systemic, because it predicts that recovery of isolates or genotypes will be clustered on particular plants. However, clustering can also be ascribed to variation in susceptibility among plants.

Phylogenetic analysis of a subset of isolates sampled from dandelion in Netherlands showed that *B. cinerea* and *B. pseudocinerea* were the predominant species. The latter is a recently described species which is morphologically very similar but phylogenetically distinct from *B. cinerea* (Walker et al., 2011). Based on the morphology of the isolates sampled from a spectrum of plants in the UK, it may be assumed that these also mostly represent *B. cinerea* and *B. pseudocinerea*, although this would need extensive sequence analysis to be confirmed. The phylogenetic analysis also identified two isolates of distinct *Botrytis* species belonging to clade 2 (Staats et al., 2005), one of which (DAN39) is presumably from *B. mali*, a postharvest pathogen of apple with an as yet poorly explored distribution (O'Gorman et al., 2008). It is perhaps relevant to note that this isolate was sampled from a dandelion plant growing in a grass patch adjacent to an apple and pear orchard of an organic farm. The second isolate from *Botrytis* clade 2, DAN5, was related but not identical to *B. caroliniana* and *B. fabiopsis*, and might represent a novel *Botrytis* species. An earlier study by Shipunov et al. (2008) on endophytic fungi in *Centaurea stoebe* identified, besides *B. cinerea*, six distinct phylogenetic groups of *Botrytis* isolates. Four of these groups were closely related to *B. cinerea* and one of them might represent *B. pseudocinerea* (which had not yet been described at the time of the study by Shipunov et al., 2008). The isolates bot079 and bot378 reported in Shipunov et al. (2008), however, were very distant from the clade containing *B. cinerea* and grouped with *B. paeoniae*, alike the *B. mali* isolate DAN39. Unfortunately the isolates from *C. stoebe* are no longer available for further phylogenetic comparison (A. Shipunov, personal communication). The above results and those of Shipunov et al. (2008) demonstrate that several new *Botrytis* species can be retrieved as endophytes from a single host in a limited sampling. A more extensive phylogenetic analysis of *Botrytis* isolates from multiple hosts and locations would likely reveal many more novel species. If more extensive sampling and further phylogenetic analysis would indeed confirm a greater diversity of *Botrytis* species than previously appreciated (Staats et al., 2005; Hyde et al., 2014), it raises the question as to how many times the cryptic life-style has arisen.

As with all endophytic organisms, the question arises as to how the host defense system and the pathogen are interacting (Schulz and Boyle, 2005; Newton et al., 2010). There are a number of possibilities. First, the fungus may evade the host defense system by not producing signals which activate defenses strongly enough to destroy the fungus. This is consistent with the increased infection frequency in unshaded lettuce plants in experiment 6: rapidly photosynthesizing plants are likely to have more nutrients in the apoplast, and thus allow more pronounced growth if they do not also have better defense. It is likely that *Botrytis* produces little or no phytotoxic metabolites and proteins, or plant cell wall degrading enzymes, while it displays symptomless internal infection behavior. If it did produce such compounds, as *Botrytis “*normally” does (reviewed by van Kan, 2006), this would result in irreversible damage to plant cells and culminate in disease symptoms. It remains elusive why *Botrytis* does not produce such damaging compounds during cryptic infection. Is it by lack of a (plant-derived) signal to activate the expression of the genes, or is the plant actively suppressing the expression of virulence genes?

An alternative view of the interaction is that host defense continually destroys *Botrytis* locally, but establishment in new areas is frequent enough to maintain the fungus associated with the plant. In experiment 6, spray inoculation with *B. cinerea* spores incurred a 20% reduction in growth, without altering infection density. This reduction in growth might reflect the cost of effective defense against a forceful attack; this defense evidently does not alter the balance between systemic infection and host defense. The similarity in frequency of cryptic infection in plants which did and did not have seed-borne infection removed by fungicide in experiment 5 again points to a balanced system in which too much local growth can be eliminated by the host. The hypothesis of a balanced growth/elimination system implies that previously infested plant tissues might become uninfested, as well as the reverse. We have no data on this. Experiments to address this question would be complex because of the destructive nature of current methods to detect infection.

The spore density at the point of first encounter is likely a crucial determinant in whether the interaction becomes asymptomatic or necrotic. At high spore density, and in the presence of nutrients, the fungus can rapidly and efficiently germinate (van den Heuvel, 1981) and produce a spectrum of phytotoxic metabolites and proteins at sufficient concentrations to cause death of multiple plant cells (van Kan, 2006) and set necrotic development in motion. Causing host cell death is in essence a physiologically irreversible decision, although there are cases where primary necrotic lesions fail to develop into an expanding lesion, possibly due to an effective restriction of the fungus by the host (Benito et al., 1998; Coertze and Holz, 2002). In order to achieve symptomless *Botrytis* infection in plants in our experiments, it was important to start the infection with a low inoculum dosage. The low number of spores and concomitant low density of germlings on the plant surface might not release sufficient phytotoxic metabolites and proteins to activate the plant cell death machinery, and thereby fail to kill cells that would act as an entry point for the fungus. Failure to induce cell death would force the fungus to enter the plant through natural openings and grow in the plant interior where it can retrieve nutrients. The growth of the fungus inside the plant would need to remain restricted and be synchronized with that of the host, possibly by mechanisms similar to those reported for *Epichloë* endophytes(Tanaka et al., 2012).

A final possible view of the interaction in this system is that the infection is in some circumstances beneficial to the host plant, so that specific signals from *Botrytis* lead to over-riding of normal defense signals. A hint that this might be so is given by the positive relation between lettuce growth and the frequency with which *Botrytis* could be recovered from the plants in experiment 7, presumably representing the density of infection. The nature of such signaling processes can only be conjectured at present. Recent studies by Weiberg et al. (2013) described how small RNAs of *Botrytis cinerea* can actively silence host genes that are involved in immunity, and thereby promote virulence of the pathogen. Conversely, it is conceivable that small RNAs from a host plant could silence *Botrytis* genes that are required for the activation of virulence mechanisms, and so prevent the production of damaging fungal proteins and metabolites. In the fungal grass endophyte *Epichloë*, the symbiotic interaction depends on an intact reactive oxygen signaling and MAP kinase signaling. The disruption of components of either of these signaling processes results in breakdown of symbiosis and switch to disease development (Tanaka et al., 2012).

The economic importance of this form of infection could be considerable in a susceptible crop such as lettuce, cyclamen or *Primula* × *polyantha* in which persistent infections last for the lifetime of the crop, causing disease and loss at maturity or after harvest. The importance of the demonstration of a large reservoir of infested vegetation in the wider environment depends mainly on how much inoculum it supplies. If this is sufficiently low, there could be no economic implications. On the other hand, given the number of plant species shown to harbor infection, infections in natural vegetation could be an important source of conidia into otherwise clean protected environments. It is less clear what could be done about it, but it suggests that effective integrated management of *Botrytis* should assume that inoculum is present not only in the air but probably latent in the crops. The best management strategies will focus on the physiological state of the plant and the intrinsic resistance or tolerance of the host to necrotic disease development.

If we can understand the mechanism that determines whether the interaction becomes symptomless or necrotic, it might be possible to steer the interaction in one or other direction, by modifying the environmental conditions, or by applying chemicals that trigger a switch in lifestyle. This might result in active suppression of necrotic development of *Botrytis*, or in the forced interruption of the symptomless infection.

## Author contributions

MS and JvK coordinated the sampling, designed and supervised the experiments, and wrote the manuscript. CE, DE, RT, AS, DT, and ME performed the experiments. CE prepared Figure 1, RT prepared Figure 2, DE prepared Figure 4.

### Conflict of interest statement

The authors declare that the research was conducted in the absence of any commercial or financial relationships that could be construed as a potential conflict of interest.

## Supplementary material

Figure S1 **Phylogenetic position of 47 *Botrytis* isolates from asymptomatic dandelion (labeled by DAN and a number) in the genus based on HSP60 sequences**. Sequences of recognized *Botrytis* species are taken from Hyde et al. (2014), sequences from *B. mali* were determined by O'Gorman et al. (2008), the sequence of isolate BOT079 was determined by Shipunov et al. (2008). Phylogenetic tree construction used a maximum likelihood method with 1000 bootstraps. Only bootstrap values higher than 50% are displayed at the nodes.

Figure S2 **Phylogenetic position of 47 *Botrytis* isolates from asymptomatic dandelion (labeled by DAN and a number) in the genus based on G3PDH sequences**. Sequences of recognized *Botrytis* species are taken from Hyde et al. (2014), sequences from *B. mali* were determined by O'Gorman et al. (2008). Phylogenetic tree construction used a maximum likelihood method with 1000 bootstraps. Only bootstrap values higher than 50% are displayed at the nodes.

Figure S3 **Phylogenetic position of 13 *Botrytis* isolates from asymptomatic dandelion (labeled by DAN and a number) in the genus based on RPB2 sequences**. Sequences of recognized *Botrytis* species are taken from Hyde et al. (2014), sequences from *B. mali* were determined by O'Gorman et al. (2008). Phylogenetic tree construction used a maximum likelihood method with 1000 bootstraps. Only bootstrap values higher than 50% are displayed at the nodes.

Figure S4 **Phylogenetic position of 16 *Botrytis cinerea* isolates from asymptomatic dandelion (labeled by DAN and a number) in the genus based on G3890 sequences**. *B. cinerea* strain B05.10 serves as the reference and *B. calthae* strain MUCL2830 was used as an outgroup. Phylogenetic tree construction used a maximum likelihood method with 1000 bootstraps. Only bootstrap values higher than 50% are displayed at the nodes.

Figure S5 **Phylogenetic position of 16 *Botrytis cinerea* isolates from asymptomatic dandelion (labeled by DAN and a number) in the genus based on FG1020 sequences**. Sequences from *B. cinerea* strain B05.10 and *B. pseudocinerea* strain VD110 (determined by Leroch et al., 2013) serve as the reference while *B. calthae* strain MUCL2830 was used as an outgroup. Phylogenetic tree construction used a maximum likelihood method with 1000 bootstraps. Only bootstrap values higher than 50% are displayed at the nodes.

Figure S6 **Phylogenetic position of 14 *Botrytis* isolates from asymptomatic dandelion (labeled by DAN and a number) in the genus based on MRR1 sequences**. Sequences from *B. cinerea* strain B05.10, *Botrytis* N11SE08 and *B. pseudocinerea* strain VD256 (determined by Leroch et al., 2013) serve as the references. Phylogenetic tree construction used a maximum likelihood method with 1000 bootstraps. Only bootstrap values higher than 50% are displayed at the nodes.

Table S1 **Sequences of primers used in this study**.
