# Supplementary figures and images for: Analysis of Cryptic, Systemic Botrytis Infections in Symptomless Hosts
Source: Front Plant Sci. 2016 May 10;7:625. doi: 10.3389/fpls.2016.00625 (PMC4861902; doi:10.3389/fpls.2016.00625)

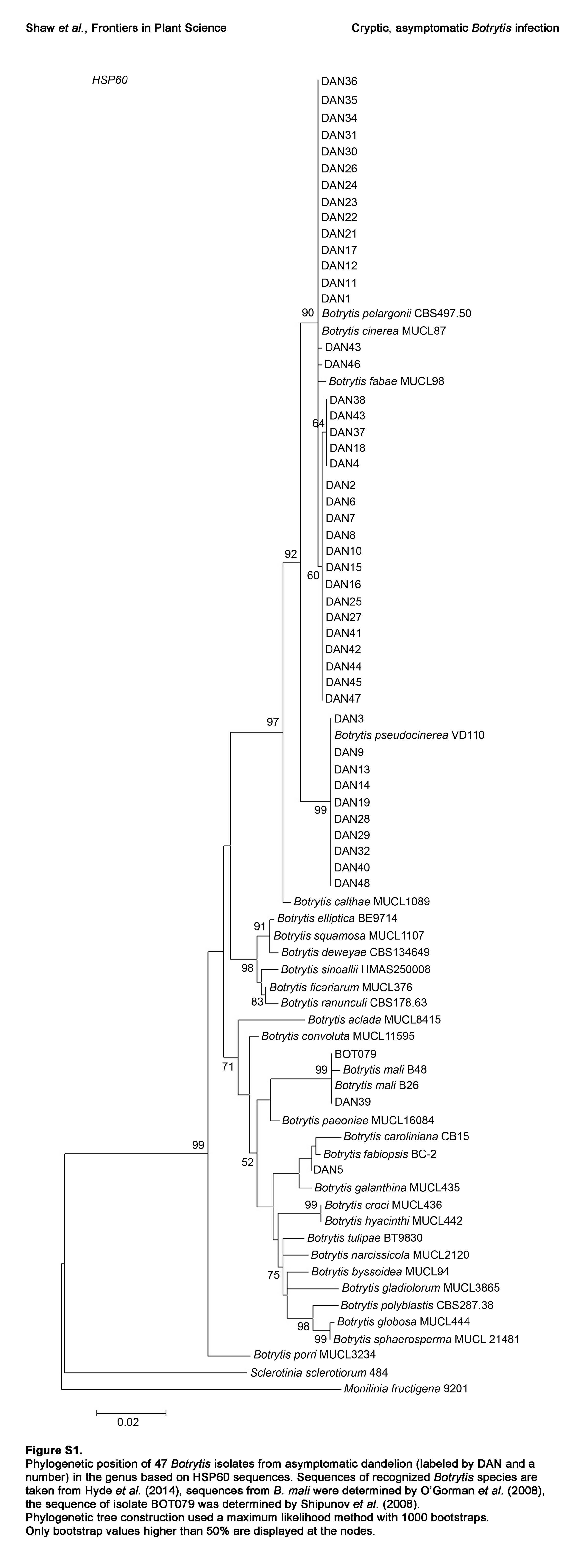

Supplement: Figure S1 — Phylogenetic position of 47 Botrytis isolates from asymptomatic dandelion (labeled by DAN and a number) in the genus based on HSP60 sequences. Sequences of recognized Botrytis species are taken from Hyde et al. (2014), sequences from B. mali were determined by O'Gorman et al. (2008), the sequence of isolate BOT079 was determined by Shipunov et al. (2008). Phylogenetic tree construction used a maximum likelihood method with 1000 bootstraps. Only bootstrap values higher than 50% are displayed at the nodes. [file Image1.TIF]

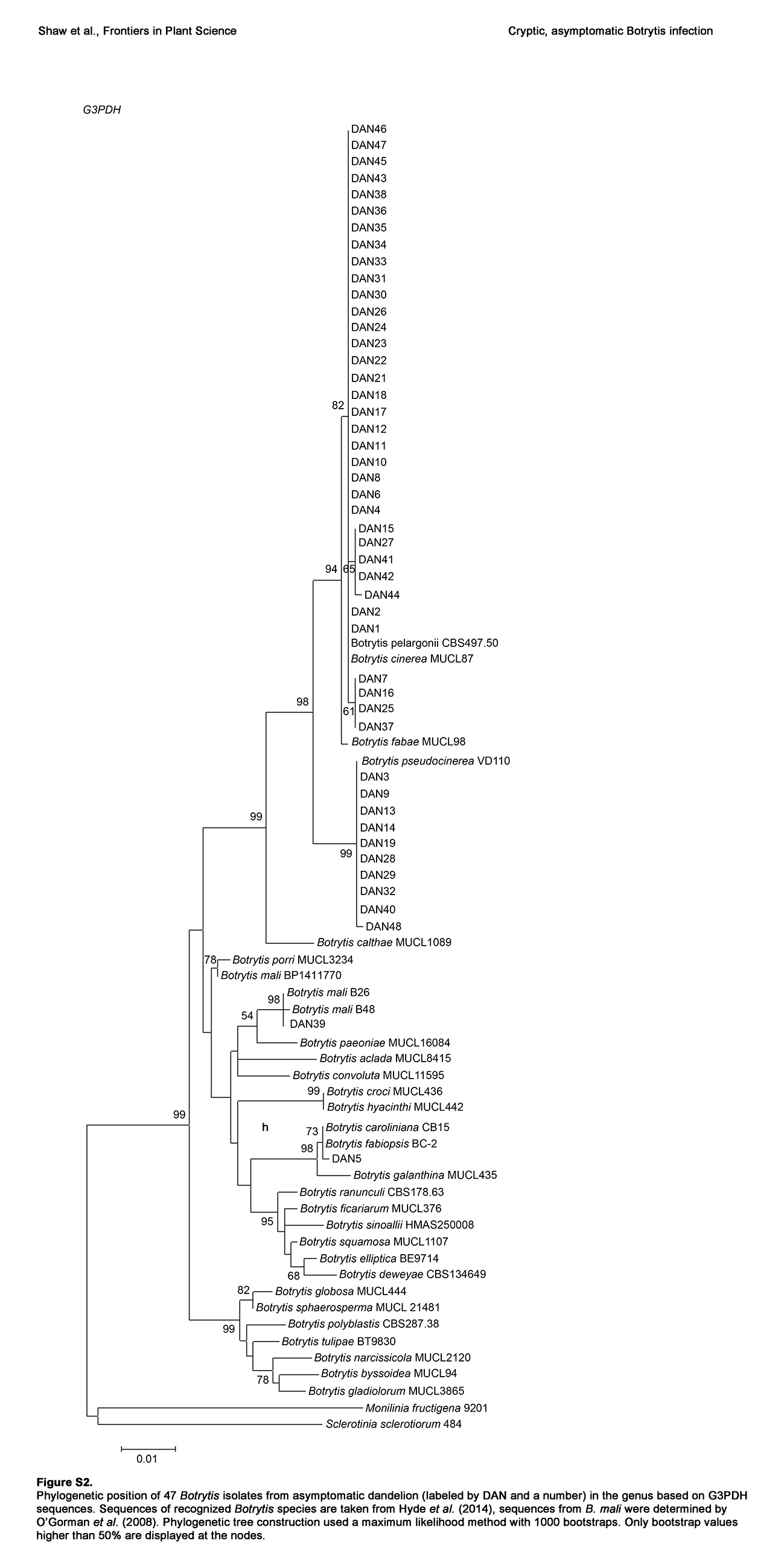

Supplement: Figure S2 — Phylogenetic position of 47 Botrytis isolates from asymptomatic dandelion (labeled by DAN and a number) in the genus based on G3PDH sequences. Sequences of recognized Botrytis species are taken from Hyde et al. (2014), sequences from B. mali were determined by O'Gorman et al. (2008). Phylogenetic tree construction used a maximum likelihood method with 1000 bootstraps. Only bootstrap values higher than 50% are displayed at the nodes. [file Image2.TIF]

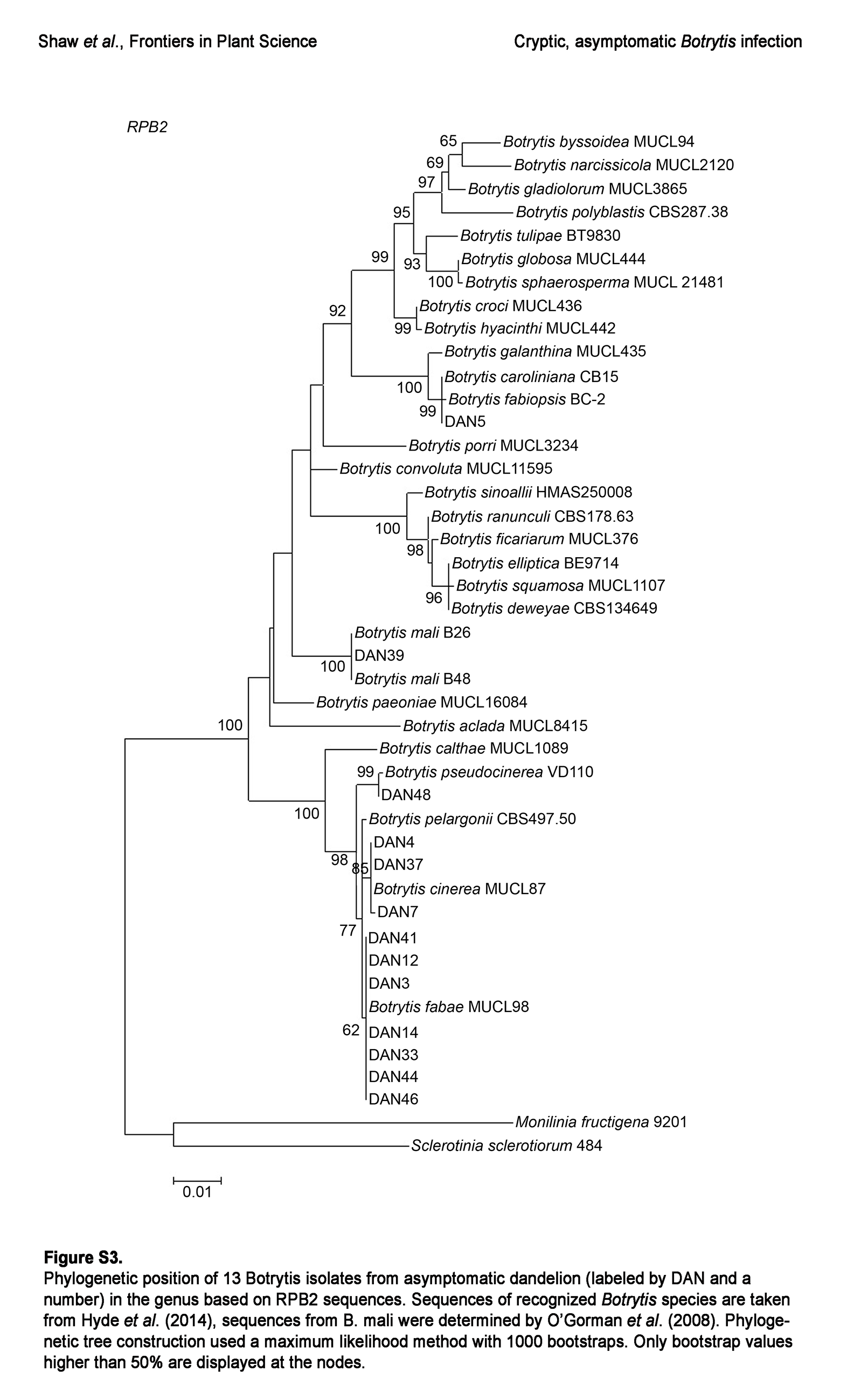

Supplement: Figure S3 — Phylogenetic position of 13 Botrytis isolates from asymptomatic dandelion (labeled by DAN and a number) in the genus based on RPB2 sequences. Sequences of recognized Botrytis species are taken from Hyde et al. (2014), sequences from B. mali were determined by O'Gorman et al. (2008). Phylogenetic tree construction used a maximum likelihood method with 1000 bootstraps. Only bootstrap values higher than 50% are displayed at the nodes. [file Image3.TIF]

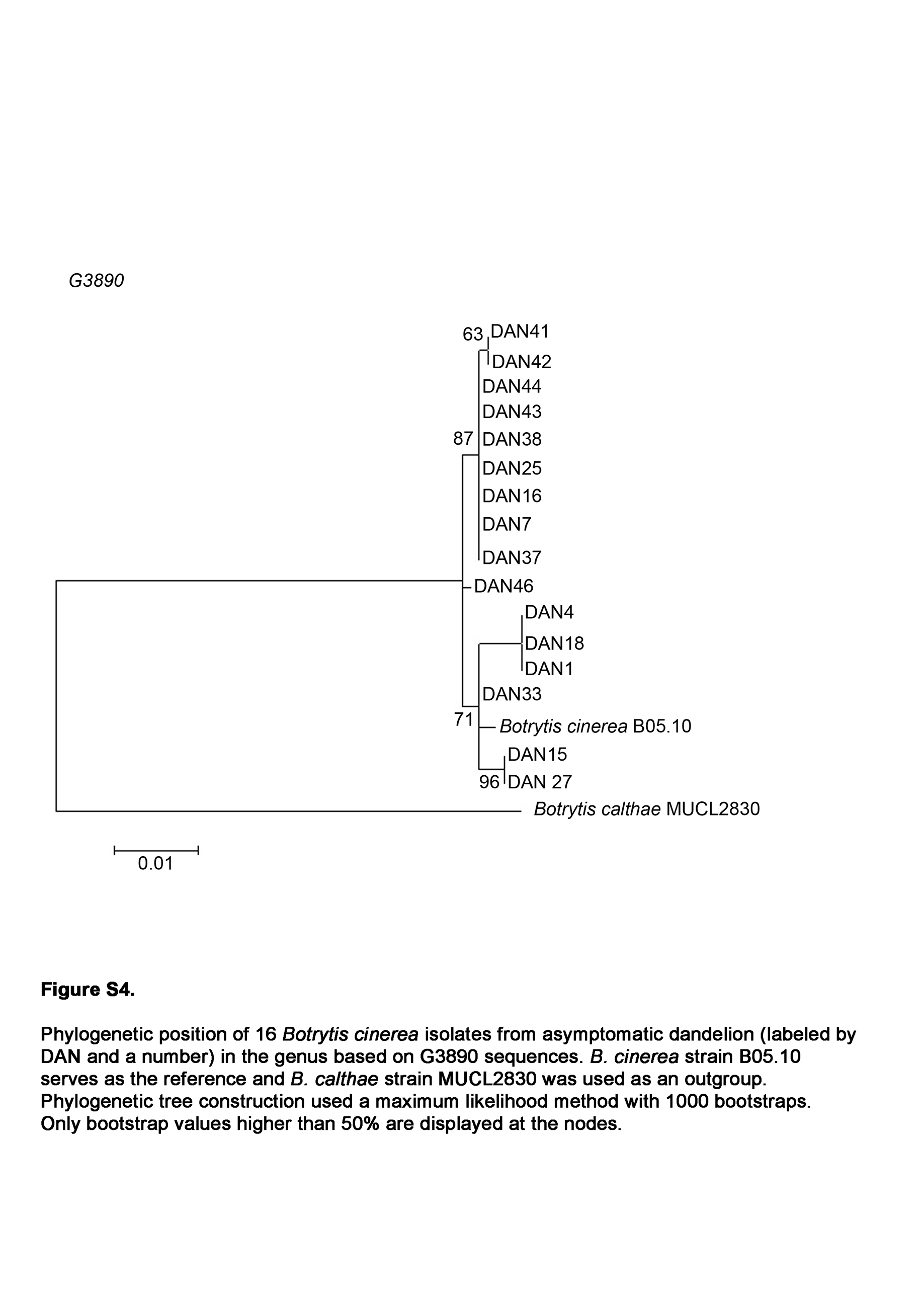

Supplement: Figure S4 — Phylogenetic position of 16 Botrytis cinerea isolates from asymptomatic dandelion (labeled by DAN and a number) in the genus based on G3890 sequences. B. cinerea strain B05.10 serves as the reference and B. calthae strain MUCL2830 was used as an outgroup. Phylogenetic tree construction used a maximum likelihood method with 1000 bootstraps. Only bootstrap values higher than 50% are displayed at the nodes. [file Image4.TIF]

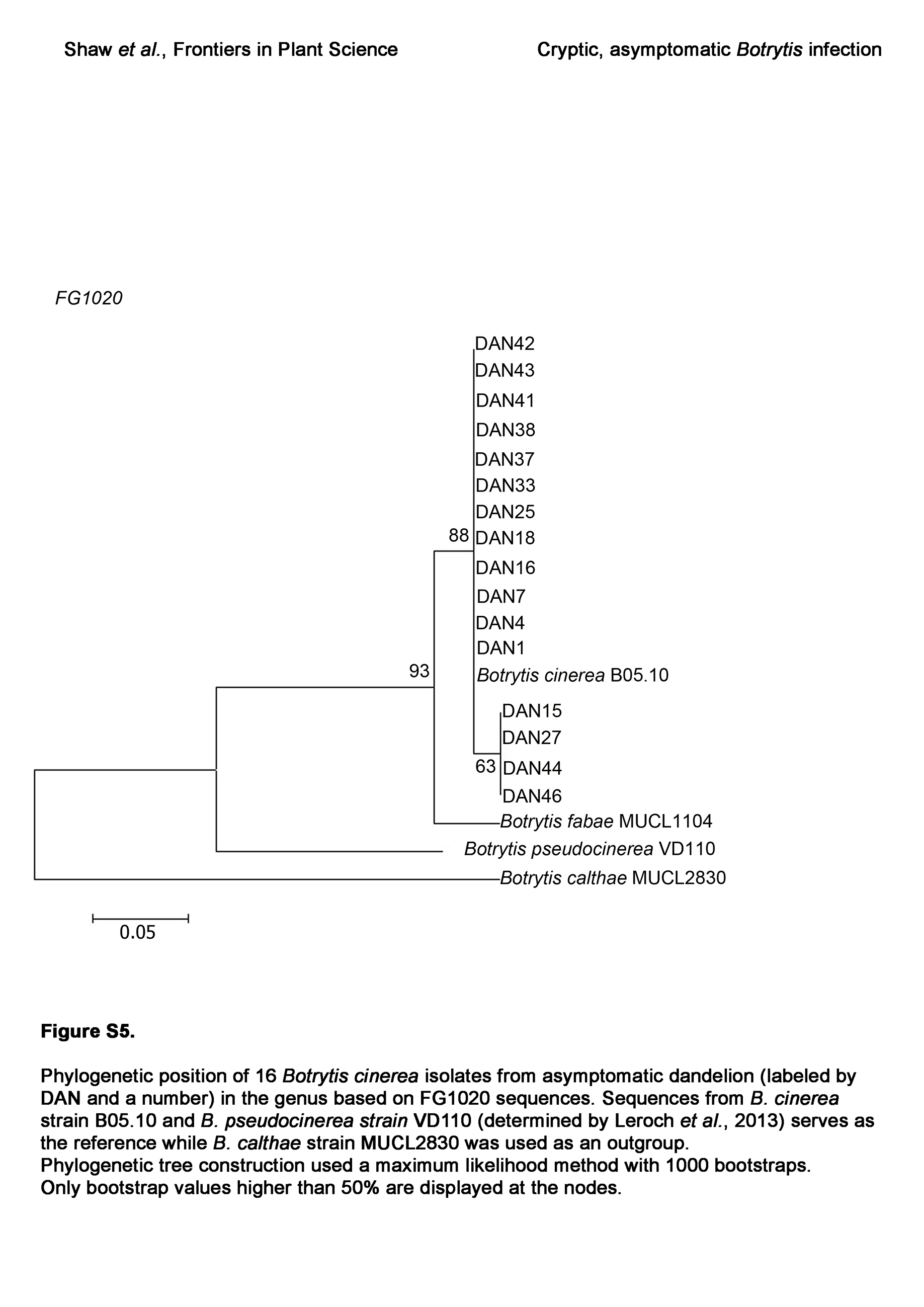

Supplement: Figure S5 — Phylogenetic position of 16 Botrytis cinerea isolates from asymptomatic dandelion (labeled by DAN and a number) in the genus based on FG1020 sequences. Sequences from B. cinerea strain B05.10 and B. pseudocinerea strain VD110 (determined by Leroch et al., 2013) serve as the reference while B. calthae strain MUCL2830 was used as an outgroup. Phylogenetic tree construction used a maximum likelihood method with 1000 bootstraps. Only bootstrap values higher than 50% are displayed at the nodes. [file Image5.TIF]

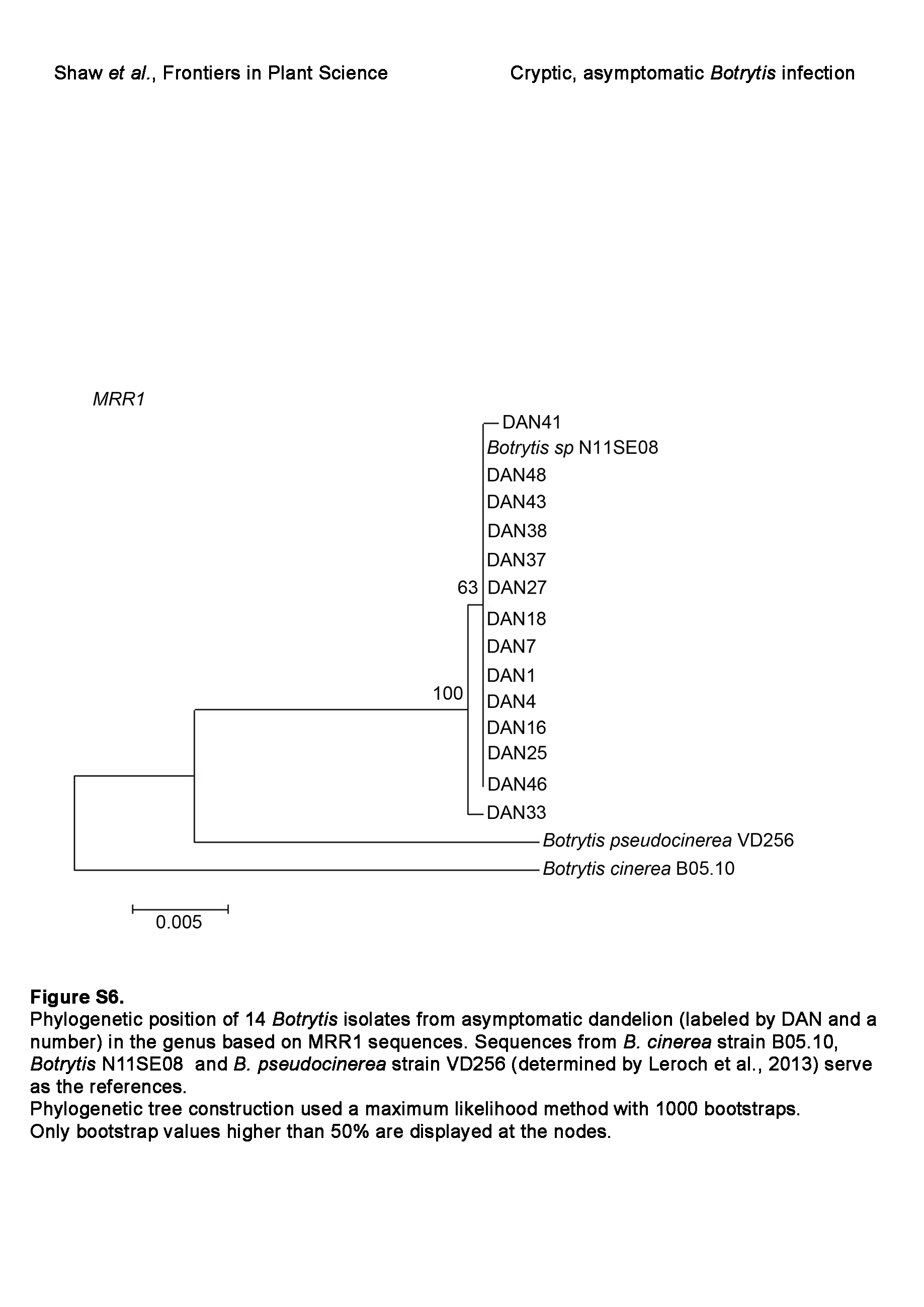

Supplement: Figure S6 — Phylogenetic position of 14 Botrytis isolates from asymptomatic dandelion (labeled by DAN and a number) in the genus based on MRR1 sequences. Sequences from B. cinerea strain B05.10, Botrytis N11SE08 and B. pseudocinerea strain VD256 (determined by Leroch et al., 2013) serve as the references. Phylogenetic tree construction used a maximum likelihood method with 1000 bootstraps. Only bootstrap values higher than 50% are displayed at the nodes. [file Image6.TIF]
